# A Systematic Review on the Usability of Web-Based Applications in Advocating Consumers on Food Safety

**DOI:** 10.3390/foods11010115

**Published:** 2022-01-03

**Authors:** Wen-Li Seow, Umi Kalsom Md Ariffin, Sook Yee Lim, Nurul Azmawati Mohamed, Kai Wei Lee, Navin Kumar Devaraj, Syafinaz Amin-Nordin

**Affiliations:** 1 Department of Medical Microbiology, Faculty of Medicine and Health Sciences, Universiti Putra Malaysia, Serdang 43400, Malaysia; wenlyseow@gmail.com (W.-L.S.); documi78@gmail.com (U.K.M.A.); l.sookyee@yahoo.com (S.Y.L.); 2 Faculty of Applied Sciences, UCSI University, Cheras, Kuala Lumpur 56000, Malaysia; 3 Faculty of Medicine and Health Sciences, Universiti Sains Islam Malaysia, Persiaran Ilmu, Bandar Baru Nilai, Nilai 71800, Malaysia; drnurul@usim.edu.my; 4 Department of Pre-Clinical Sciences, Faculty of Medicine and Health Sciences, Universiti Tunku Abdul Rahman, Kajang 43000, Malaysia; lee_kai_wei@yahoo.com; 5 Department of Family Medicine, Faculty of Medicine and Health Sciences, Universiti Putra Malaysia, Serdang 43400, Malaysia; knavin@upm.edu.my

**Keywords:** food safety, consumers, restaurants, web-based education, knowledge

## Abstract

Food safety is an important indicator of public health, as foodborne illnesses continue to cause productivity and economic loss. In recent years, web-based applications have been extensively used by the online users’ population. Almost one third (28.3%) of online users found web-based application to be a notable source of food safety information. The objective of the current review is to determine the effectiveness of a web-based application systems as a health promotion tool for consumers to increase their knowledge and awareness of food safety. A systematic literature review was conducted by analyzing 11 selected web-based food safety education-related articles. The studies were categorized into several themes: (1) web-based applications used in accessing food safety information; (2) food safety evaluation and perception among consumers; (3) beliefs and level of knowledge, attitude and practices (KAP) of consumers; and (4) impact and contribution of social media use. A diverse number of online applications have been utilized to promote food safety education among consumers, yet these web-based applications need to be improved with regards to social connection and integration among consumers. KAP surveys were conducted on the majority of the respondents with a particular focus on their knowledge level. Findings show that web-based applications may act as an alternative to the traditional media in enhancing food safety education among consumers, especially youths who are tech-savvy.

## 1. Introduction

Foodborne diseases are preventable, yet they are still a significant cause of public health concern that lead to economic burden. Foodborne illness outbreaks are commonly associated with restaurants and households. Pathogenic bacteria and antimicrobial-resistant bacteria which are commonly found in collected cooked food and ready-to-eat food samples lead to foodborne diseases. Therefore, case studies involving microbiological investigation of food samples from consumers who suffered from food poisoning after eating at restaurants and their own homes have been studied worldwide [1,2,3,4,5,6] to reduce the risk of food poisoning and even death among consumers. The knowledge, attitude and practice (KAP) among consumers acts as foremost elements in promoting knowledge and awareness of food safety, to which the increase in food safety knowledge level positively contributes in terms of encouraging better attitudes, as well as relatively affecting food safety practices [7,8]. 

However, due to the current lockdown all around the world due to the COVID-19 pandemic, people are spending more time cooking at home. Nevertheless, it is still very convenient for people to consume outside food offered by restaurants, food stalls and food courts during the pre- and post-COVID-19 pandemic periods, due to the increasing use of online applications by restaurants to help consumers make their dining options. Reports have shown that an increasing number of consumers uses the Internet in seeking food services and products. However, their selections are mostly based on food quality, price and service quality; very few are concerned about food safety information on the Internet [9,10]. In addition, Internet information majorly abounds with food business advertisements that promote the best restaurants in terms of food varieties; however, websites that educate consumers on food safety at restaurants are still very limited. This indicates that consumers have limited access to knowledge that helps them to identify poor quality food that can cause food poisoning. Hence, it is vital to enhance consumers’ food safety KAP through an online food safety education strategy. Still, there are a few web-based applications used for food safety educational purposes, such as Facebook, YouTube, Reddit, Google, Yahoo!, MySpace, LinkedIn, Twitter, micro blogs, Baidu news and other websites [11,12,13,14,15,16,17,18,19,20,21]. Next, surveys, CD ROMs, Power Point presentations, lab kits, printed booklets, the Baidu Index and an online focus group were employed as educational tools on these applications. A huge number of previous systematic reviews dedicated to the positive impact of food safety educational interventions has shown an improved self-reported food safety behavior among consumers [22]. 

Additionally, nowadays, Internet users are looking for the information in real-time and focusing on human-centered engagement. When they use the application as an information sharing platform, this may create a follow-through practice among consumers [23]. For example, Internet users prefer to browse various categories of health information on the Internet before they seek a medical professional for further clarification and this helps in improving the quality of health. Bach and Wenz (2020) found that tracking one’s health, as well as general health information, is among the most popular categories searched by users [24]. It can be seen that integrated technology apps are used to promote healthy food purchasing and consumption [25]. In recent years, food consumers started to highlight the impacts and improvements of food consumption on environmental issues, food safety, human’s health and animal welfare [26,27]. Furthermore, food poisoning cases and foodborne diseases are becoming major concerns worldwide [28,29,30]. Hence, food safety information and education could become among the main topics searched on the Internet. Internet platforms have been used in food business marketing; however, the focus is mostly on promoting the varieties and popularity of the foods and beverages, rather than on food safety. Lack of food safety educational movements may lead to the emerging of novel pathogenic bacteria, toxins and antibiotic resistance. Consequently, this may lead to food contamination leading to foodborne outbreaks [31]. Thus, food safety experts should take the same initiative to promote the importance of food safety through web-based applications that can reach out to Internet users anytime anywhere. The purpose of this review is to identify the usability of a web-based application system among consumers. The research question of the current review focuses on the effectiveness of a web-based application system as a health promotion tool for consumers to increase their knowledge and awareness of food safety. 

## 2. Materials and Methods

Prior to the start of article inclusion, the study methods were documented in an international prospective register of systematic reviews (PROSPERO) protocol, registration number: CRD42020214644. The Preferred Reporting Items for Systematic Reviews and Meta-Analyses (PRISMA) guidelines were followed in conducting this systematic review and meta-analysis and in reporting its results [32]. 

### 2.1. Eligibility Criteria 

The eligibility criteria of current review, including the domain, inclusion and exclusion criteria, are stated in Table 1. 

### 2.2. Search Strategy 

Three databases were included in the current review, namely EBSCOhost, Ovid and Science Direct. Three researchers independently searched for the potential studies published in journals from the inception of the study to 31 December 2020. The search terms are shown in Table 2. Duplicates were removed, as well as studies including pediatric participants. The titles and abstracts of identified citations were screened for relevance to the review questions using a prior tested form. The researchers also identified the identified papers through forward search, which identified the studies that cited a prior identified study. Any identified relevant papers were included in current study [33]. Participants of the included studies were consumers of the restaurants, consumers who used online platforms, or consumers related to food safety education with an age range from 11 years old and above. All kinds of exposure to internet/web-based applications used for other purposes than educational, such as food safety tracking systems, advertisement apps, review papers and non-English articles were excluded. The identified papers were sorted to remove the duplicates. Next, the titles, abstract and full text assessment were performed and the studies involved children were also excluded.

### 2.3. Study Selection 

Three reviewers reviewed the titles and abstracts of the previous studies during the search. All included studies used web-based applications to access information on food safety. The web-based applications used to search for food safety information were categorized into blogs, the Baidu Index, websites, social media and search engines. Besides, the perception refers to the way food safety information is organized, interpreted and purposely experienced [34]. The included studies also evaluated the improvement in food safety belief, knowledge, attitude and practices among consumers. Knowledge is defined as the understanding level of consumers about food safety information provided, while attitude refers to the tendency and preference of consumers to react positively or negatively to food safety-related experiences. Practices are the actions of the consumers in regard to the knowledge and attitude involved in food safety matters [35]. The selected studies also included the contribution of web-based applications used in enhancing food safety education. The results of the selected studies were measured through questionnaires which were conducted among food consumers.

### 2.4. Data Extraction 

Three reviewers independently extracted data and assessed the risk of bias for each study. Reasons for exclusion and percentage of agreement between the assessors were documented. From the included articles, the following information was extracted: title, names of authors, publication year, country, ethnic origin, methods, statistical test, results and conclusion. The data were manually organized into standardized files for further data research analysis. 

### 2.5. Quality Assessment 

**Figure 1:**
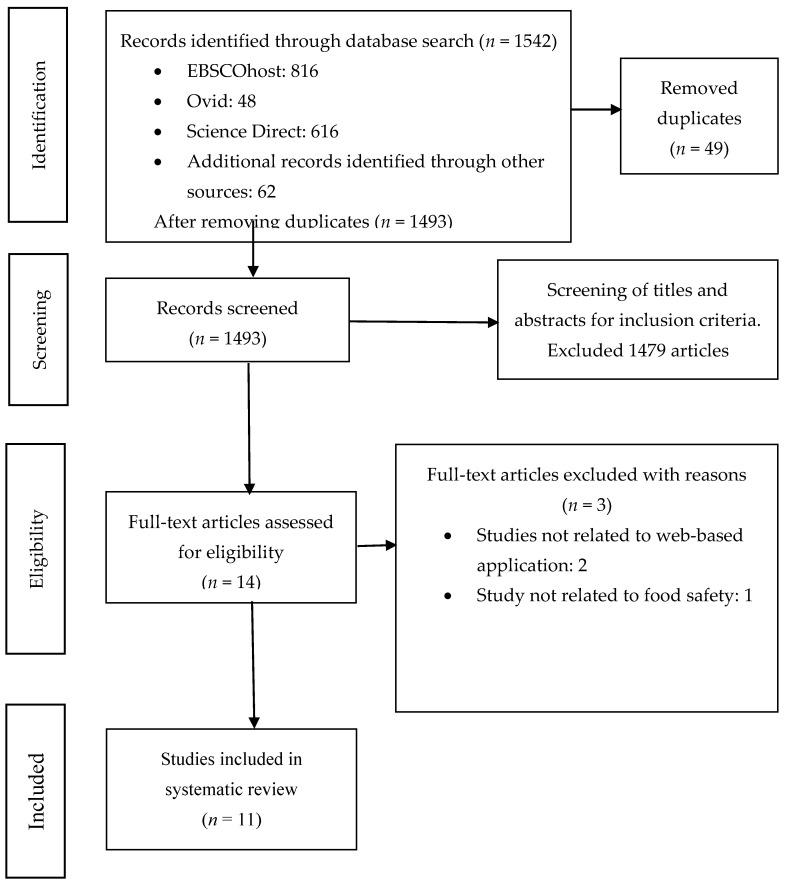
The PRISMA chart of the current review.

The risk of bias of randomized controlled trials (RCTs) was assessed using the Cochrane risk-of-bias tool for randomized trials (RoB 2.0) [36], in which five domains were evaluated: randomization process, deviations from intended interventions, missing data, outcome measurement and selection of reported results. Each domain was assessed for risk of bias. A study was graded as (1) “low risk of bias”, when a low risk of bias was determined for all domains; (2) “some concerns”, if at least one domain assessed raised some concerns, but not to be at high risk of bias for any single domain; or (3) “high risk of bias”, when a high risk of bias was reached for at least one domain, or the study judgment included some concerns in multiple domains [36]. For non-RCTs, the risk bias assessment tool for non-randomized studies (RoBANS) was used. This tool comprises six domains: selection of participants, confounding variables, measurement of invention, blinding of outcome assessment, incomplete outcome data and selective outcome reporting. [37] Each domain was assessed as “High”, “Low”, or “Unclear”. Risk of bias ratings were conducted independently by two investigators. A third reviewer was consulted in case of discrepancies between the first two reviewers. The summary of the selected studies is displayed in Figure 1. 

## 3. Results

### 3.1. General Characteristics of Studies Included for Review 

**Figure 2:**
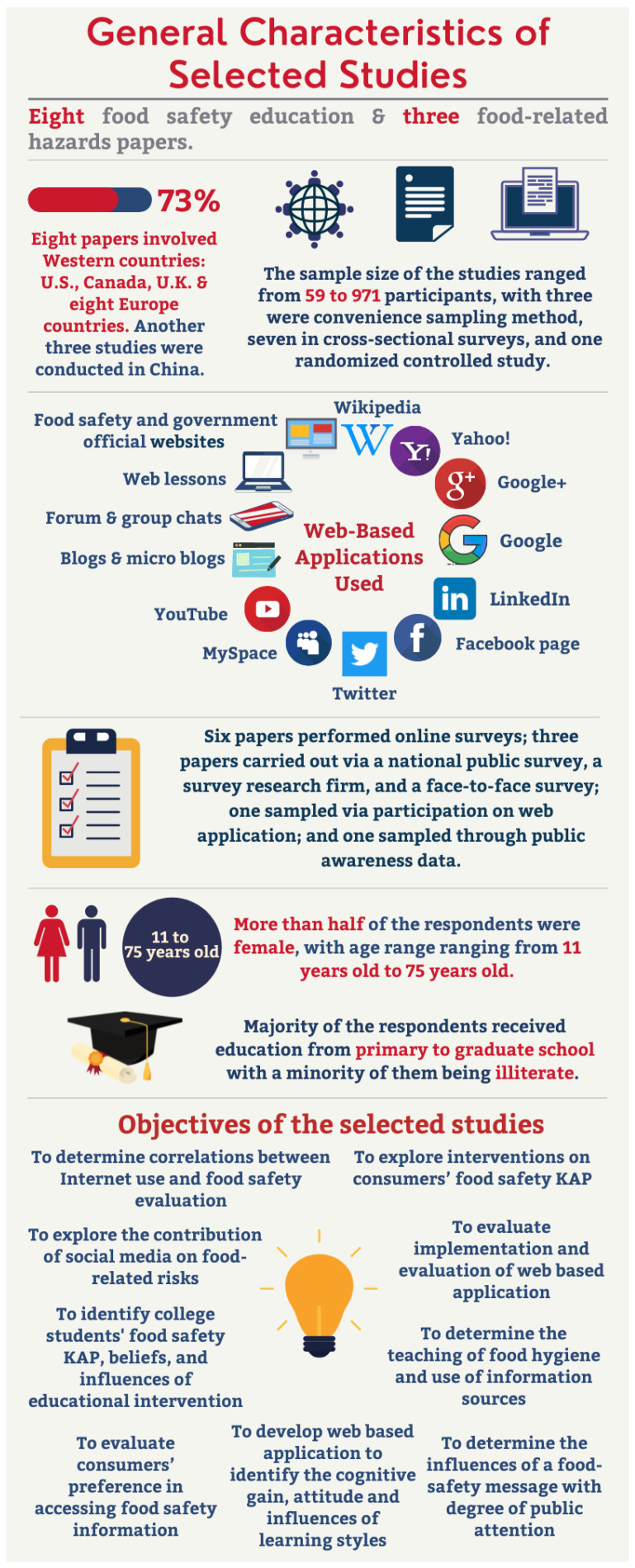
The general characteristics of the selected studies.

The characteristics of the identified articles were compared by referring to the research questions. From a total of 11 articles, 8 articles were related to food safety education and 3 others were related to food-related hazards. The studies were conducted all around the world: three were conducted in China, five in the United States, one in Canada, one in the United Kingdom and one in Europe, which involved eight countries. One study was carried out through the government national public survey; one was conducted through a survey research firm; one sampled participants via a face-to-face survey; one sampled via participation on a web application; one sampled through public awareness data; and six sampled participants through an online survey. The sample size of the 11 selected studies ranged from 59 to 10,048 participants, with another study sampled on micro blogs and news (*n* = 414,234). The convenience sampling method was performed in three studies, seven in cross-sectional surveys and one performed randomized controlled study. Five studies involved online Internet web-based applications; one engaged with several websites learning; one focused on social media and online media; one included government websites in the study; one involved micro blogs and news; and two involved web-based and print materials. Only one study specifically engaged with the Facebook page. The contents of the 11 main articles are tabulated in Table 3. The objectives of the included studies differed from the current study design, which focuses on the usability of a web-based application in advocating consumers’ food safety. One study focused on the correlations between Internet use and consumers’ food safety evaluation; three involved interventions on consumers’ food safety knowledge, attitude and practices (KAP); one focused on college students’ food safety KAP and beliefs and explored the influences of educational intervention; one involved participants’ preference in accessing food safety information; one determined the influences of a food-safety messages on the degree of public attention; one developed a web-based application to identify the cognitive gain, attitude and influences of different learning styles; one focused on the implementation and evaluation of a web-based application; one determined the teaching of food hygiene and use of information sources; and one study explored the contribution of social media to food-related risks. General characteristics of selected studies are summarized in Figure 2. 

### 3.2. Socio-Demographic Distribution among Consumers 

More than one-third (36.4%) of the studies included the socio-demographic characteristics of the respondents, namely, gender, age and education. Similar distributions and trends were observed with regards to gender, age and educational level. More than half of the respondents were female, with sample sizes ranging from 59 to 971. The age range of the respondents was very wide, ranging from 11 years old to 75 years old. The majority of the respondents received education from primary to graduate school with a minority of them being illiterate. 

### 3.3. Web-Based Applications Used in Accessing Food Safety Information 

Of the 11 selected studies, 9 studies mentioned that consumers used internet web-based applications to access information on food safety, in which a study provided interactive food safety instructional materials through web lessons [17]; 1 study created a food safety instructional website [20]; 4 studies combined the use of online web-based applications and conventional materials, such as printed materials, videos, CD-ROMs and lab kits [13,14,18,21]; 1 study focused on consumers’ perception, worry level and knowledge of food-related hazards [39]; 1 study combined the use of different websites, resources from health units and other public health inspectors [12]; 1 study specifically used Facebook as intervention to improve food safety KAP [11]; 1 study determined the access level of Internet content to browse news and to find information [38]; and 1 study searched the micro-blogs and Baidu news on the ‘‘set-style yogurt and jelly event’’ [19].

Yarrow et al. (2009) determined that the food safety beliefs and KAP among college students were improved by providing web lessons that included audio clips, flash card activities, clip art, quizzes, graphics, puzzles and drag-n-drop exercises on the Internet [17]. A web-based application that contained videos, games, activities, quizzes and lessons helped middle school students to pick up food safety knowledge, meeting various learning styles [19]. These two studies used a single channel to identify student’s food safety level. 

On the other hand, another four studies used both online media and traditional media to advocate consumers’ food safety education. One of these four studies mentioned that the teachers used different types of resources to teach food hygiene syllabuses in primary schools, such as websites, videos, CD-ROMs, printed materials and teaching packs. Resources obtained from the British Nutrition Foundation (BNF), Food Standards Agency (FSA), Food and Drink Federation (FDF), British Meat Education Service, Milk Marketing Board, government websites, BBC website and Expresso were well utilized by the teachers to teach food hygiene and cleanliness [18]. Furthermore, secondary school science teachers used CD-ROMs, PowerPoint presentations, lab kits and a website named Food Safety FIRST that had module activities of inquiry-based learning and science education standards to develop and to evaluate a food safety education program [21]. Kosa et al. (2011) determined the effectiveness of using a purpose-built website and print materials such as booklet to educate food safety practices among 272 older adults aged from 70 to 75 years old, completed pre- and post-surveys, with qualification for impact evaluation [13]. The study also found that less than half (40.0%) of the senior citizens who accessed the food safety information website found the application as being very useful and 44.0% of the participants would refer back to the website to obtain more information on food safety [13]. Next, a high percentage of the participants, i.e., 81.8%, agreed that the website assisted them in learning more about food safety and 83.9% were of the opinion that the website could be a reliable source of food safety information [13]. Kuttschreuter et al. (2014) identified that consumers (*n* = 1264) from eight countries used three information channels to seek information about fresh vegetables’ food safety risks. The participants accessed social media such as Twitter, online blogs, online forum, online chat groups, Facebook, MySpace, Linkedin, Google+ and YouTube videos to obtain information on issues related to food safety, while some accessed online media such as news websites, search engines and the official websites of food-related agencies; the third channel of traditional media included listening to radio, reading newspapers and watching television [14]. The Internet was found to be the second most frequently used platform in accessing information on food safety after the television [14]. The study also indicated that consumers with a low knowledge level used the applications less frequently than consumers with a higher knowledge level [14]. YouTube was identified as the most effective application used and videos posted on Facebook pages were also considered as the preferred way for food safety education among college students [12]. Findings obtained by Manu et al. (2021) also indicated that YouTube was a better social media educational tool compared to Facebook, Twitter and Pinterest [15].

Furthermore, two studies combined three platforms to identify the food safety risks and the needs for trusted information sources. Liu et al. (2014) measured the different platforms used by consumers to gain food safety information. Television, Internet and word of mouth among friends and relatives regarding food safety information were the top-three platforms used by the consumers. Books, magazines, radio and brochures were seldom used in searching for food safety information. Consumers equipped with higher knowledge frequently received food safety information from newspapers, magazines, books and brochures, compared to other groups. However, the uses of these three platforms were not significantly different among three different groups of consumers [39]. Pham et al. (2019) stated that, when accessing information on food safety, 83.5% of public health inspectors referred to government official websites [12] and they were unlikely to refer to or access non-governmental websites and industrial websites. Almost all (98.3%) of the participants agreed that online resources provided an easy access to receive new information on food safety. In terms of effective food safety information publicizing, health inspectors chose the online clearinghouse of web-based databases (50.8%) and email newsletters, as their preferred platforms (40.2%) [12]. Furthermore, Gilardi and Fubini (2005) identified the authentic Internet resources focusing on food safety information to be international organizations, European organizations, U.S. national organizations and databases [40]. A study carried out by Simeonea and Scarpatobs (2020) was in line with Pham et al.’s (2019), in which organic food consumers who were critical of and cared for the supply chain of the organic food would refer to official websites and channels on food safety information rather than referring to the information shared on social media [41]. Liu et al. (2014) also mentioned that only medical doctors, research institutes and consumers’ associations were considered as the most reliable information sources about food safety [39]. This could be due to the perception that, although there is an abundance of information on food safety on the Internet, the quality and reliability of the information are still very poor [39]. This can be related to the fact that consumers used the Internet to access those parties’ food safety information due to the growing usage of the Internet and online news and high participation rate by youths and middle-aged participants [39]. 

Generally, Facebook is the most common platform used by food safety authorities and researchers to evaluate consumers’ food safety level. This can be evidenced in Mayer et al.’s (2012) study, in which they mentioned that the majority of the students had a Facebook account (97.0%), 17.0% had a MySpace account, 12% had a LinkedIn account and 26% had a Twitter account. Facebook and YouTube were considered the preferable media used to search for food safety information compared to podcasts and MySpace. Videos were recommended in delivering food safety information on a Facebook page and YouTube was the most frequently used effective tool [11]. A previous study conducted by Sutter et al. (2021) also supported the study carried out by Mayer and Harrison (2012), i.e., a huge number of food safety education information posts, such as food parenting posts and questions looking for answers, were shared on Facebook and Reddit [16]. In addition, more than half of the participants (from 62.0% to 67.0%; from 51.0% to 53.0%) were likely to use the food safety website links given and Facebook in the future to learn about food safety. Although Manu et al. (2021) also mentioned that Facebook and Twitter were good platforms to share one’s thoughts and opinions, part of the findings contrasted with the studies mentioned earlier which claimed that Facebook and Twitter were not considered as effective educational tools to engage professional parties and companies, since college students used social media to connect with their personal lives and not for knowledge-enhancement purposes [15]. To enhance engagement and interactions among consumers, YouTube was considered a competent social media tool to discuss food safety topics [11,15], while, in terms of social search websites, Wikipedia, YouTube, Yahoo! and Answers were very popular among the search engines [14]. Another two studies’ consumers used the Internet and we media to find food safety information. Zhang et al. (2019) indicated that consumers used the Internet to browse Weibo and news and to search for food safety information [38]. While Peng et al. (2015) applied three groups of data: number of Baidu news, the Baidu Index and releasing and forwarding numbers of microblogs to identify public awareness of the “set-style yogurt and jelly event’’ [19]. Table 4 shows the web-based applications used to access food safety information.

### 3.4. Food Safety Evaluation and Perception among Consumers 

Generally, food safety evaluation among consumers in China can be considered good. When asked to identify the most common food hazards among consumers, the consumers listed spurious food and low-quality food as the first and second food hazards of concern and food treated with pesticides and decayed food as the third and fourth food hazards of concern. Surprisingly, genetic modified food and food additives were perceived as lowest in risk and in causing worries. Consumers also perceived medical doctors, research institutions, consumers’ associations and the government as the most reliable sources of information pertaining food safety. Consumers also perceived themselves as those who cared the most about their health, while food producers were considered as the least concerned party with regards to consumers’ health and were associated to the country’s food safety controversy [39]. Furthermore, Peng et al. (2015) also indicated that recent food safety issues occurred in China affected the public’s trust level in the food industry, in that consumers were hugely affected by the public opinions announced by “key opinion leaders” (KOL) on micro-blogs, even though the suspected food industry proclaimed the issue was not true. The declamation did not regain consumers’ trust in consuming food products [19]. In addition, teachers in a UK primary school perceived that the guidance on key food hygiene messages would benefit both teachers and parents [18]. A study conducted by Zhang et al. (2019) indicated that participants with a higher educational level, women and urban participants were more worried about food safety compared to men and those residing in rural areas [38]. Participants who lived in the eastern and more developed territories of China, who were younger than 31 years old and frequent Internet users were also found to be more concerned about food safety issues. Frequent Internet users showed a higher negative perception of “very unsafe” and “unsafe” in food safety evaluation and the evaluation of food safety concerns increased from 2013 to 2015 [38]. Specifically, participants with a higher frequency of Internet use in searching for information through the Weibo search engine had a lower food safety evaluation. The study also found that Internet users with an education level lower than senior high school level had a stronger negative evaluation of food safety, while people with happier lives had a higher food safety evaluation [38]. In addition, an intervention study showed that the participants strongly agreed that the risk of contracting foodborne illnesses increased as age increased [13].

### 3.5. Food Safety Beliefs, Knowledge, Attitude and Practices among Consumers 

The intervention study conducted by Yarrow et al. (2009) changed college students’ food safety beliefs. The students’ belief that, by following safe food handling practices, washing hands before cooking and finishing cooked food that has been unrefrigerated in less than two hours, the chances of becoming sick would be reduced. Students also reviewed that eating or handling perishable food, such as raw vegetables and sprouts, raw red meat, raw white meat and raw shellfish, could be risky to body health. Students became less concerned with the risks of eating raw fruits. They also became concerned with home-prepared food as the primary source of foodborne illness and their belief that food microorganism contamination was more serious than previously recognized increased [17]. 

Regardless of their knowledge level, Liu et al. (2014) mentioned that 60.1% of consumers possessed a high level of food safety knowledge and were concerned about food-related hazards, with 21.7% of the consumers considering having a moderate knowledge level on food safety and another 18.2% a poor knowledge level [39]. Consumers who had higher food safety knowledge were women and those from urban areas. Although consumers were considered to be very knowledgeable about spurious food and low-quality food, they were least equipped with knowledge on genetically modified food and food additives. In addition, although the Internet was frequently used by consumers, it showed no significant difference in the consumers’ knowledge level [39]. A study conducted by Qiang et al. (2011) contradicted the findings obtained by Liu et al. (2014), showing that China residents gained food safety knowledge through food safety news published in websites that characterized potential hazards of violated food products [42]. A total of 237 participants were noticeably confident in their food safety knowledge of proper hand washing (91.1%), cross contamination (87.7%) and time–temperature abuse (83.9%) [12]. Moreover, the majority of the participants were also very confident in food pathogen knowledge, namely, *Salmonella* (53.8%), *E.coli* 0157:H7 (52.7%) and *Campylobacter* (46.8%). A study conducted by Gruenfeldova et al. (2019) recorded that almost all participants (98.0%) knew about Salmonella, followed by 90.0% with knowledge of *E. coli* and 79.0% of *Staphylococcus aureus*, while only 58.0%, 71.0% and 72.0% knew about *Campylobacter*, *Listeria monocytogenes* and *Bacillus cereus*, respectively [43]. Woh et al. (2016) also found that participants were little equipped with knowledge of foodborne pathogens *Salmonella* (3.7%), *E. coli* (1.0%), *B. cereus* (1.0%), *Vibrio* (0.5%) and *S. aureus* (0.3%) [44]. The finding agrees with the study by Pham et al. (2012), who found that online training participants showed great enhancement in food safety knowledge, for example, relative to the fact that unhygienic hands are the main source of food contamination, or the need to throw away torn chopping boards and clean kitchen sinks before and after use [45]. Yarrow et al. (2009) also mentioned that college students’ knowledge scores increased after the intervention. Students were aware that they should not prepare food for others when they had diarrhea and they knew that cooking eggs until fully firm could kill pathogens. For hamburger patties, students learned that patties should be cooked to an internal temperature of 160 °F and non-pink patties are considered as not fully safe to eat [17]. Mayer and Harrison (2012) claimed that the knowledge level of the college students who received intervention showed no significant difference during the pre-test [11]. Generally, participants who frequently cooked (from four to six times per week) had higher food safety knowledge than those who cooked from one to three times per week [11]. The finding agreed with the study performed by Gruenfeldova et al. (2019), in that the cooking time spent by the respondents had a direct effect on their food safety knowledge level [43]. Moreover, only 16.0% of the respondents could name the 14 food allergens, with only 51.0% being able to recognize more than 7 allergens, namely, gluten (82.0%), nuts (80.0%), milk (79.0%) and mollusks (30.0%) [43].

The attitudes of the participants were considered good, whereby 93.6% of them believed that they were responsible for making sure the food they ate was clean and safe. In total, 89.2% of the participants had negative attitudes as they considered that safety was the issue to them [11]. Furthermore, the intervention group showed a significant improvement in the attitude scores, indicating that the food safety education implemented had a positive influence on the participants’ attitudes [11]. Yarrow et al.’s (2009) finding was also in line with Mayer and Harrison’s (2016), in that the attitudes of college students showed significant changes during intervention. Students from health and non-health groups were concerned (*p* ≤ 0.007) about non-refrigerated food such as beans and rice, having unfirmed egg yolks, drinking unpasteurized fruit juices such as apple juice, eating ready-to-eat hotdogs, eating alfalfa sprouts and thawing perishable food on the kitchen bench [17]. In addition, secondary school students and teachers also showed positive attitudes about using the Food, Flies and Fungus web-based food safety program. The students preferred the flexibility provided by the program, in that they could use the program in the classroom, library or computer room. The website was easy to use and fun, as the students enjoyed the videos, games and activities of the program. The teachers also preferred to use the program, as it assisted them to achieve food safety educational objectives and it fitted the existing course [20]. Eleanor et al. (2019), on the other hand, claimed that food safety education could be also enhanced when consumers are equipped with a positive attitude toward food safety, thus reducing the risk of foodborne diseases [45]. 

The practices of the participants had significantly changed as they consumed less cold deli meats and hot dogs without heating, undercooked meat and poultry, refrigerated smoked seafood, unpasteurized juice and raw sprouts [13]. Although the practice behaviors of the college students showed no significant difference during the pre-test, the practice scores showed an improvement after the intervention; they agreed to change their behaviors before and after the intervention by increasing the frequency of hand washing, separating raw poultry and meats from other groceries, using different cutting boards for different raw food materials and always keeping leftovers in the fridge [11]. Woh et al. (2016) also mentioned that the participants did bathe regularly (96.96%) and used soap and clean running water to wash their hands (86.4%). Nevertheless, only 9.1% of the participants went for a medical checkup once in six months [44]. Moreover, for both health and non-health major college students, surprisingly, only health major students showed positive changes for self-reported practices. This could be seen by the fact that health major students would avoid preparing food for others when they had diarrhea, would choose to use a thermometer when cooking and would not leave refrigerated food at room temperature for a long period [17]. Beffa-Negrini et al. (2007) also mentioned that middle school teachers showed improvements on personal food safety habits relative to hand-washing methods and fruit-washing habits before eating. The teachers also functioned as role models to the students as the teachers were confident in sharing proper food-handling practices in front of the students [21]. On ‘‘set-style yogurt and jelly event’’ issues, consumers would stop consuming rumored food products and would look for substitute products when they received the news propagated by opinion leaders on micro-blogs. Hence, drops in sales figures and switches in brand loyalty could further oblige the food industry to improve their food safety standard operational procedures (SOP) and corporate responsibility [19].

### 3.6. Impact and Contribution of Social Media Use in Enhancing Food Safety Education

The rapid increase in the world’s Internet use and participation of young and middle-aged consumers have increased the access rate and knowledge of food safety through interpersonal social media platforms [39]. Videos posted on social media platforms such as Facebook and YouTube act as complementary information channels to traditional media in promoting food safety education and cultivating consumers’ knowledge, attitude and practices, as all the participants owned Facebook accounts [11]. A study by Kuttschreuter et al. (2014) also shared similar findings, i.e., social media could act as an alternative to traditional media in enhancing food safety education among consumers [14]. However, a study by Kosa et al. (2011) found that food safety educational websites and printed materials had no impact on participants’ eating behaviors, although the participants began to consume less cold deli meats [13]. The use of a website as an effective food safety information application has helped in enhancing consumers’ food safety knowledge, as findings showed that 72.0% of the participants who accessed the website learned at least one new food safety knowledge item [13]. Beffa-Negrini et al. (2007) determined that secondary school teachers were equipped in critically evaluating online food safety information on the Internet through the Food Safety FIRST program and the teachers were confident in teaching food safety concepts. The program also aided teachers in integrating food safety into science instruction and helped teachers design class lessons [21]. Furthermore, an appealing food safety website would increase the frequency of website usage and consequently increase students’ learning achievements [20]. Online educational modules also showed a positive impact on health major students’ food safety knowledge, whereby all scores increased right after intervention [17]. A summary of the six selected studies is presented in Table 3, Table 4 and Table 5, showing web-based applications used in accessing food safety information and food safety KAP among consumers, respectively. 

## 4. Discussion

The aim of the current review is to identify the effectiveness of a web-based application system as a health promotion tool for consumers to increase their knowledge and awareness of food safety. The findings of the selected previous studies are divergent; however, they can generally be separated into three main criteria, namely, use of web-based applications, food safety perceptions among consumers and the beliefs and KAP level of food safety among consumers. 

Participants involved in the selected previous studies were diversified in terms of their age and study area coverage. Compared to other family members, parents act as the proactive characters who usually take good care of the family’ food consumption; hence, they are more concerned about the health of the family members. As food safety issues are important to them, this encourages them to find out more about food safety education through online activities. Parents usually utilized social media platforms such as Facebook and Reddit to search for reliable information in providing safe and nutritious food for their children [16]. Thus, it is clear that parents act as vital characters in society to encourage family members to practice food safety at home [45]. Furthermore, teachers could also play an important role in providing food safety knowledge to students at different educational levels [18,20,21]. Teachers who have gone through food safety interventions might ignite students’ interests in learning food safety [21]. 

In general, consumers with a higher education level had higher comprehension and could make rational judgments when seeking food safety information. This is possibly due to the fact that educated consumers have the ability to search for online resources as they are exposed to a broad range of online resources. In addition, educated consumers with a high income can pay for and acquire higher mass media exposure; therefore, they are frequently exposed to food safety issues propagated by web-based applications. This socio-demographic group is also associated with a higher level of concerns toward food safety, namely, food quality, food safety regulations and socially related food safety risks [46]. This can be proved by the fact that higher educated college students, especially health-related students, showed improvement in their beliefs, attitude, knowledge and practices right after a food safety intervention [17]. 

The number of Internet users has been growing worldwide during the pre- and post-COVID-19 pandemic and young Internet users, in particular, who are very familiar with social media, may use applications as an information channel to receive information. Hence, the development of an advanced information system and AI applications is especially important in developing food safety databases. The database–website search engine system is a constructive tool in assisting consumers in seeking food safety information and compare food poisoning case data. It is also a credible and reliable tool to search for food safety information from central and local authorities [47]. 

The selected 11 previous studies highlighted the use of web-based applications in providing and spreading food safety information. However, integration and social interactions were rarely discussed in the papers. In general, social media were categorized into information, entertainment, personal identity, integration and social interactions [43]. Recent approaches such as web-based and social media have shown good improvement in propagating food safety information; however, the integration and social interactions among consumers are still lacking and need to be improved. This is because connection among consumers helps motivate consumers’ involvement in effective food safety education and enhances KAP in food safety in the long term. Furthermore, a web-based application advocates a learning environment that promotes social interactions on food safety information among consumers. 

On the other hand, food safety education, in previous studies, focused on the KAP concept. In terms of food safety knowledge, topics on foodborne illness were considered as an essential topic. The knowledge of cross-contamination, microbiological foodborne pathogens and hand hygiene were sufficient and included in most research studies to evaluate participants’ food safety knowledge [43,48,49,50,51]. Surprisingly, only a few researchers included, in their research studies, the topic of food allergens and used it to evaluate participants’ food safety knowledge [42]. Thus, the knowledge of food allergens should be highlighted by researchers in terms of embracing food safety education standards and connecting with consumers in different regions. The best web-based food safety education platform has yet to be well established; thus, high impact studies will help its development. 

In addition, the current finding shows that the majority of the selected studies conducted before year 2010 combined both traditional and web-based learning materials, in that CD ROMs, offline videos and printed materials were used. Technology evolved in the 2010s and 2020s, with more people using web-based applications to surf for information and food safety education through web-based platforms has been gradually acknowledged by the authorities and consumers. Hence, food safety knowledge management needs to be promoted through higher education institutions and shared among policy makers and consumers. 

The strengths of this systematic review include the use of the PRISMA statement and adherence to a registered research protocol. During the study selection process, a detailed search strategy was used across many databases with a broad date range and strict inclusion criteria. To our knowledge, this is the first review to determine the effectiveness of a web-based application system as a health promotion tool for consumers to increase their knowledge and awareness of food safety. Moreover, the current review also highlights the importance of web-based applications in advocating food safety education through the sharing of food safety information and the lack of effectiveness in connecting and promoting interactions among consumers through online applications. Although it is obvious that social media act as a vital application in the sharing of food safety information, traditional media such as newspapers and TV are still practical in disseminating food safety information to consumers. It is also worth noting that this systematic review does have several limitations. First, the search strategy was restricted to papers written in English; thus, this resulted in an underestimation of the amount of evidence, since papers written in other languages were excluded. Furthermore, some of the selected studies had a limited sample size and used the non-random sampling procedure, which would limit the generalizability of the results. Another limitation is that the selected previous studies did not focus on studying food safety education through web-based applications among restaurants’ consumers and comparing it to consumers who cook at home. More case studies, intervention studies and controlled studies related to the impacts of web-based applications are needed for future research. 

## 5. Conclusions 

In conclusion, the findings of the current review indicate that web-based applications can be utilized as a health promotion tool for consumers to increase their beliefs, knowledge, attitude and practices relative to food safety—especially their knowledge level. Websites, blogs, social media and search engines are commonly used by the consumers for searching for food safety information. Furthermore, it proves that an increase in web-based application use may increase the consumers’ food safety beliefs and KAP, especially in their knowledge level. Compared to traditional printed educational materials, web-based applications can also be considered as an alternative platform to promote food safety education among consumers.

## Figures and Tables

**Table 1:** Domain, inclusion and exclusion criteria.

Domain	Inclusion Criteria	Exclusion Criteria
Publication year	Studies published between 1 January 2010 and 31 December 2020.	Studies published before 1 January 2010 and after 31 December 2020.
Publication type	Original research studies, case studies, short reports, letters, methodologies and other publications that were published in scholarly journals.	Review papers, short reports, letters, methodologies and other publications that were not published in journals.
Research design	Quantitative studies of intervention, cross-sectional, or cohort studies.	Qualitative studies such as interview, focus groups, or case studies.
Language	Studies that included the full text in the English language.	Non-English studies were excluded.
Targeted population	Population that was either engaged in food safety web-based application or involved in food safety education.	Population that was neither engaged in food safety web-based applications nor involved in food safety education.
Targeted group	Early adolescents who were 11 years old and above.	Early adolescents who were below 11 years old.
Study area	(1) Included the web-based education on food safety at restaurants, cafés, food courts, school canteens, food establishments and home-based. (2) Web-based applications included blogs, the Baidu Index, websites, social media and search engines. (3) The study area also included the combined exposure with any other conventional educational method, such as television, newspaper, posters and others.	All kinds of exposure to internet/web-based applications used for other purposes than food safety education, such as food safety tracking systems, prevalence studies of microorganisms and advertisement apps.

**Table 2:** Search terms.

Search Category	Search Term
Food safety	“food safety education” OR “restaurant” OR “food premise” OR “consumer” OR “customer” OR “knowledge” OR “awareness” OR “perception”
Web-based	“web based” OR “internet” OR “application”

**Table 3:** Characteristics and key findings of the studies on usability of a web-based application.

Author (s)	Topic/Purposes	Location	Social/Online Media Type	Sample	Data Collection	Key Findings
Zhang et al., 2019 [38]	* FS, perception and evaluation	China	Internet use/All	China Social Survey (CSS) (*n* = 10,048)	Cross-sectional survey	Negative correlations between Internet use and consumers’ food safety evaluation among rural residents, young people and less educated residents.
Kosa et al., 2011 [13]	FS, intervention, practices	United States	Web-based, print materials	From web-enabled panel (*n* = 566)	Randomized controlled study	Small improvements were observed among the groups. However, the difference in the changes between the two groups was not significant. Although print materials were relatively accepted, the educational materials did not have an impact on participants’ behavior.
Liu et al., 2014 [39]	FS, perception	China	Internet use/All	Public at supermarkets, malls, residential areas and parks (*n* = 971)	Quantitative survey of convenient sampling	High degree of concerns and a moderate knowledge of food-related hazards among consumers, in which television, the Internet, radio and word of mouth were not significantly different among consumers.
Pham et al., 2012 [12]	FS, perception and needs	Canada	Government website	Public health inspectors in Ontario (*n* = 239)	Cross-sectional online survey	The public health inspectors’ preference in accessing food safety information was through government websites (83.5%), “talk to other PHI” (66.9%) and in-house resources (44.8%).
Mayer and Harrison 2016 [11]	FS, intervention of * KAP	United States	Facebook	College students (*n* = 710)	Convenience sampling	The intervention contributes to improving food safety attitudes, practices and knowledge through a Facebook page. Participants who spent more time on the Facebook page showed improvement in attitudes and practices.
Kuttschreuter et al., 2014 [14]	FS, evaluation	Belgium, Germany, Ireland, Italy, Portugal, Spain, The Netherlands, United Kingdom	Social media and online media	Public recruited by market research agencies (*n* = 1262)	Cross- sectional survey	Social media is a compatible information media, but it could not replace traditional and online media. Participants who tended to use the online or offline media were motivated to find additional information and were responsive to food-related risks.
Beffa-Negrini et al., 2007 [21]	FS education, intervention	United States	Website	Secondary school science teachers (*n* = 38)	Cross-sectional online modules	The three-module intervention indicated that the teachers were intended to teach FS and they were comfortable in teaching FS. The teachers were also confident in carrying out FS lessons by answering FS questions and teaching this topic. The students also were interested in FS and the teachers were confident that the FS concepts taught would meet national science standards.
Lynch et al., 2007 [20]	FS education, intervention	United States	Website	Middle-school students (*n* = 217)	Convenience sampling	The students’ knowledge was increased from pre-test to post-test. However, the sixth-grade students had lower improvement than other students. This web-based application also met students’ different learning styles and they enjoyed using the website.
Yarrow et al., 2009 [17]	FS education, intervention	United States	Web lessons	College students (*n* = 59)	Cross-sectional online modules	Three-module web lessons intervention was able to improve the attitude, belief and knowledge scores of both majors. There was an increase in the attitude and practices among health majors, with better intervention results.
Peng et al., 2015 [19]	FS issues, awareness, purchase behavior	China	We media (micro blogs and Baidu news)	Three groups of data (*n* = 414,234)	Cross-sectional sampling	Three groups of data: numbers of related releasing and forwarding micro blogs, number of Baidu news and the Baidu Index were used to evaluate consumers’ awareness and purchase behavior on the ‘‘set-style yogurt and jelly event’’ reported. The results indicated that we media increased the propagation of opinion leaders’ thoughts and initiated a mass discussion on food safety messages with the public.
Bielby et al., 2006 [18]	FS education	United Kingdom	websites	Primary school teachers (*n* = 875)	Cross-sectional sampling	Hand washing (96.0%) and personal hygiene (90.0%) were the common principles taught in schools. Furthermore, the most frequent (98.0%) methods used to teach food hygiene included teachers talking about food hygiene and carrying out practical activities. Lack of kitchen facilities and science laboratories as well as limited curriculum time were limitations to teaching food hygiene. Moreover, the most frequent resources used to teach food hygiene were posters (98.0%) and worksheets (93.0%), while websites (82.0%) were ranked sixth.

* FS, food safety; * KAP, knowledge, attitude and practices.

**Table 4:** Web-based applications used to access food safety information.

Reference(s)	Category of Web-Based Applications	Type of Web-Based Application(S)
[13,18,20,21]	Website	Food safety websites
[12]		Government official websites
[14]		Wikipedia
[14,19]		News
[17]		Web lessons
[11,14,15]	Social media	Facebook
[14,15]		MySpace, Linkedin, Twitter
[11,14,15]		YouTube
[14,19]		Blogs, micro blogs
[14,15]	Search engine	Google, Yahoo!

**Table 5:** Food safety KAP among consumers.

Reference(s)	Belief and KAP Level	Description
[17]	Belief	The students’ belief that, by following safe food handling practices, washing hands before cooking and finishing cooked food that has been unrefrigerated in less than two hours, the chances of becoming sick would be reduced.
[42]	Knowledge	The consumers were most concerned about spurious food and low-quality food but did not know much about (were less equipped in terms of) genetically modified food and food additives.
[42]		The Internet had no significant impact on consumers’ knowledge level.
[17]		There was an increase in food safety knowledge among consumers after using web-based applications.
[12]		Food pathogens’ knowledge was evaluated among consumers.
[11]		An increase in cooking frequency resulted in an increase in food safety knowledge.
[11,17,20]	Attitude	Food safety education encouraged consumers’ positive attitudes and vice versa.
[11,13,17,19,21]	Practices	The food safety education implemented improved consumer’s practices toward better food safety habits.

## Data Availability

Data from this study is contained within this article.
